# Emergency department utilization for gastrointestinal care and patient characteristics associated with hospital admission in a national cohort

**DOI:** 10.1093/gastro/goad045

**Published:** 2023-07-27

**Authors:** Kelly Suchman, Nina Kohn, Arvind J Trindade

**Affiliations:** Division of Gastroenterology, Zucker School of Medicine at Hofstra/Northwell, Long Island Jewish Medical Center, New Hyde Park, NY, USA; Institute of Health Innovations and Outcomes Research, Feinstein Institutes for Medical Research, Northwell Health, Manhasset, NY, USA; Division of Gastroenterology, Zucker School of Medicine at Hofstra/Northwell, Long Island Jewish Medical Center, New Hyde Park, NY, USA; Institute of Health Innovations and Outcomes Research, Feinstein Institutes for Medical Research, Northwell Health, Manhasset, NY, USA

## Introduction

Gastrointestinal diseases and care in the USA account for a significant amount of healthcare resources and spending, recently totaling >$119 billion dollars annually [1]. In addition to ambulatory sites and clinics, a substantial portion of gastrointestinal care occurs through the emergency department (ED). Though the overall rate of ED visits in the USA has remained stable over the past 10 years [2], the number of visits for gastrointestinal complaints has been increasing [1, 3], contributing to rising healthcare costs.

Minimal studies have been published examining the burden and disposition of patients presenting to the ED with gastrointestinal complaints. One study found that gastrointestinal illnesses accounted for >10% of US ED visits in 2007, with 21.6% of patients ultimately being admitted to the hospital [3].

Given the increasing number of gastrointestinal ED visits, the goal of this study was to examine the burden of patients presenting to the ED with gastrointestinal complaints and to determine characteristics associated with these patients being admitted to the hospital.

## Materials and methods

Data were collected from the National Hospital Ambulatory Medical Care Survey (NHAMCS) [4]. This survey is conducted annually to study ambulatory care and ED services. Using a multistage sampling design, non-institutional, non-federal hospitals across the 50 states and the District of Columbia are included to create a nationally representative sample. Information collected includes patient demographics, procedures, medications, imaging, and diagnosis. All data are publicly available and de-identified.

ICD-10 discharge diagnosis codes corresponding to gastrointestinal complaints were compiled (Supplementary Table 1). NHAMCS data for the years 2016 through 2019 were collected, including discharge diagnosis, patient age, day of presentation, residence, race and ethnicity, insurance status, imaging in the ED, and disposition. Patients were included if their primary discharge diagnosis matched one of the gastrointestinal ICD-10 codes.

The association between discharge from the ED and each categorical explanatory factor was examined using the Rao–Scott chi-square test. The association between discharge and age was examined using a linear model. Multivariable logistic regression was used to examine the association between disposition (admission vs discharge) from ED and age, weekend vs weekday, residence, race and ethnicity, insurance, and imaging. For all analyses, a weighted analysis was used to account for the NHAMCS sampling design. Associations were considered significant if the *P*-value was <0.05. The analysis was performed using SAS software, version 9.4 (SAS Institute Inc., NC, USA).

## Results

A total of 565,192,302 patients presented to the ED between 2016 and 2019, 62,152,996 with gastrointestinal complaints (11.0% of total ED visits). In total, 10,320,420 (16.6%) of the patients presenting with gastrointestinal complaints were admitted to the hospital and 51,832,576 (83.4%) were discharged. Patient characteristics between those admitted and discharged can be found in Supplementary Table 2. There was a statistically significant difference between age, sex, residence, radiology procedures, and insurance (all *P *<* *0.05). Discharge diagnoses can be found in Supplementary Table 3. Abdominal pain (41.70%) and nausea/vomiting (14.89%) were the most common discharge diagnoses.

Associations between disposition and patient characteristics were examined on multivariate analysis (Table 1). Male sex (odds ratio [OR] 0.646, *P *<* *0.001), homelessness (OR 0.338, *P *=* *0.025), and nursing home residence (OR 0.494, *P *=* *0.029) were associated with hospital admission. Patient age was also associated with admission (OR 0.972, *P *<* *0.001) and the odds of admission increased by 13.0% with every 5-year age increase. Patients receiving a computed tomography scan, magnetic resonance imaging, or ultrasound in the ED were also less likely to be discharged (OR 0.516, 0.154, 0.524, respectively, all *P *<* *0.001). Race/ethnicity was associated with disposition as well (*P *=* *0.003) and patients identifying as non-Hispanic Black were more likely to be discharged (OR 1.546, *P *=* *0.001).

**Table 1.** Multivariate analysis of patient characteristics and disposition

Variable	Overall *P*-value	Item	Odds ratio^a^	95% CI	*P*-value
Sex	<0.001	Female	1 (reference)	–	–
		Male	0.646	0.557–0.749	<0.001
Day of week	0.869	Weekday	1 (reference)	–	–
		Saturday or Sunday	0.974	0.805–1.180	0.790
Patient residence	0.022	Private residence	1 (reference)	–	–
		Blank/unknown	0.760	0.386–1.499	0.428
		Homeless/homeless shelter	0.338	0.131–0.873	0.025
		Nursing home	0.494	0.262–0.930	0.029
		Other	1.116	0.533–2.338	0.771
Race/ethnicity	0.003	Non-Hispanic White	1 (reference)	–	–
		Hispanic	1.155	0.866–1.540	0.326
		Non-Hispanic Black	1.546	1.204–1.987	0.001
		Non-Hispanic Other	0.969	0.643–1.460	0.881
Insurance	0.138	Private insurance	1 (reference)	–	–
		All sources of payment are blank/unknown	0.763	0.514–1.132	0.178
		Medicaid or CHIP or other state-based program	0.877	0.689–1.117	0.288
		Medicare	0.803	0.605–1.064	0.126
		Worker’s comp/self-pay/no charge/charity/other	1.270	0.896–1.800	0.178
Computed tomography scan	<0.001	No	1 (reference)	–	–
		Yes	0.516	0.438–0.609	<0.001
Magnetic resonance imaging	<0.001	No	1 (reference)	–	–
		Yes	0.154	0.063–0.374	<0.001
Ultrasound	<0.001	No	1 (reference)	–	–
		Yes	0.524	0.406–0.675	<0.001
Other imaging	0.096	No	1 (reference)	–	–
		Yes	0.548	0.269–1.113	0.096
Age	<0.001	Age	0.972	0.966–0.977	<0.001

CHIP, Children’s Health Insurance Program; CI, confidence interval.

a An odds ratio of <1 with a significant *P*-value indicates a statistical association of being admitted to the hospital; an odds ratio of >1 with a significant *P*-value indicates a statistical association of being discharged from the emergency room.

## Discussion

Our large national cohort study confirms that gastrointestinal complaints are still a major source of utilization of the emergency room. Gastrointestinal complaints accounted for 11.0% of total ED visits between 2016 and 2019, and 16.6% of these patients were admitted to the hospital.

Though >62 million patients presented to the emergency room with gastrointestinal complaints, less than one-fifth of these patients were admitted. Prior studies have found similar findings [3]. The low admission rate shows potential opportunities for patients to receive care in ambulatory centers rather than the ED.

Our study found many associations with final disposition (hospital admission vs discharge). For example, we found that male gender was associated with admission. It is unclear whether this reflects overutilization of care vs previously documented gender disparities in healthcare [5]. The study design does not address causes for these associations. We found similar associations for residence (nursing home and homelessness). Presumably these patients are sicker with limited access to care. Increasing age was, not surprisingly, a risk factor for admission, as older age is associated with worsening co-morbidities [6].

Our study found that non-Hispanic Black patients were more likely to be discharged from the ED, though the study is not designed to account for the cause of this association. Prior studies have shown healthcare disparities in the Black population compared with other races. Inferior treatment and survival of Black Americans have been documented in gastrointestinal malignancies, including colorectal [7], gastric [8], and pancreatic [9]. Black patients also have higher rates of public insurance or being uninsured compared with non-Hispanic White patients [10]. The higher rate of discharge in this population may also be attributed to discrepancies in access to ambulatory care. Further research is needed to discern the etiology of this association.

Our study has limitations. The retrospective design has inherent limitations and does not allow for etiologies of associations found. Furthermore, the data set does not permit searching by patients’ admitting complaint, preventing us from categorizing patients by this variable. However, the large data set does provide useful information that can stimulate research into these associations.

In conclusion, gastrointestinal complaints account for a significant portion of ED visits. Only a fraction of these patients are admitted to the hospital. Further public health research is required to address this discrepancy and to examine associations found in this large national cohort study.

## Supplementary Material

goad045_Supplementary_Data
