# Supplementary material for: Emergency department utilization for gastrointestinal care and patient characteristics associated with hospital admission in a national cohort
Source: Gastroenterol Rep (Oxf). 2023 Jul 27;11:goad045. doi: 10.1093/gastro/goad045 (PMC10375061; doi:10.1093/gastro/goad045)
Supplement: goad045_Supplementary_Data [file goad045_supplementary_data.docx]

**Supplementary Table 1**. ICD-10 diagnosis codes

| Combined categories | Description | ICD-10 |
| --- | --- | --- |
| GI Malignancies | Malignant neoplasm of esophagus | C15 |
|  | Malignant neoplasm of stomach | C16 |
|  | Malignant neoplasm of small intestine | C17 |
|  | Malignant neoplasm of colon | C18 |
|  | Malignant neoplasm of rectosigmoid junction | C19 |
|  | Malignant neoplasm of rectum | C20 |
|  | Malignant neoplasm of anus and anal canal | C21 |
|  | Malignant neoplasm of liver and intrahepatic bile ducts | C22 |
|  | Malignant neoplasm of gallbladder | C23 |
|  | Malignant neoplasm of other and unspecified parts of biliary tract | C24 |
|  | Malignant neoplasm of pancreas | C25 |
|  | Malignant neoplasm of other and ill-defined digestive organs | C26 |
|  | Gastrointestinal stromal tumors | C49.A |
| GERD and esophageal diseases | Heartburn | R12 |
|  | Esophagitis | K20 |
|  | Gastro-esophageal reflux disease | K21 |
|  | Other diseases of esophagus | K22 |
|  | Disorders of esophagus in diseases classified elsewhere | K23 |
| Ulcers | Gastric ulcer | K25 |
|  | Duodenal ulcer | K26 |
|  | Peptic ulcer, site unspecified | K27 |
|  | Gastrojejunal ulcer | K28 |
|  | Gastritis and duodenitis | K29 |
| Dyspepsia | Functional dyspepsia | K30 |
| Ileus and obstruction | Paralytic ileus and intestinal obstruction without hernia | K56 |
| Diverticular diseases | Diverticular disease of intestine | K57 |
| Functional motility disorders | Irritable bowel syndrome | K58 |
|  | Other functional intestinal disorders | K59 |
| Diseases of anus and rectum | Fissure and fistula of anal and rectal regions | K60 |
|  | Abscess of anal and rectal regions | K61 |
|  | Other diseases of anus and rectum | K62 |
|  | Hemorrhoids and perianal venous thrombosis | K64 |
| Liver diseases | Hepatomegaly and splenomegaly, not elsewhere classified | R16 |
|  | Unspecified jaundice | R17 |
|  | Ascites | R18 |
|  | Alcoholic liver disease | K70 |
|  | Toxic liver disease | K71 |
|  | Hepatic failure, not elsewhere classified | K72 |
|  | Chronic hepatitis, not elsewhere classified | K73 |
|  | Fibrosis and cirrhosis of liver | K74 |
|  | Other inflammatory liver diseases | K75 |
|  | Other diseases of liver | K76 |
|  | Liver disorders in diseases classified elsewhere | K77 |
| Diseases of biliary tract and gallbladder | Cholelithiasis | K80 |
|  | Cholecystitis | K81 |
|  | Other diseases of gallbladder | K82 |
|  | Other diseases of biliary tract | K83 |
| Pancreatic diseases | Acute pancreatitis | K85 |
|  | Other diseases of pancreas | K86 |
|  | Disorders of gallbladder, biliary tract and pancreas in diseases classified elsewhere | K87 |
| Malabsorption | Celiac, tropical sprue, pancreatic steatorrhea, whipple | K90 |
| Abdominal pain | Abdominal and pelvic pain | R10 |
| Nausea and vomiting | Nausea and vomiting | R11 |
| Dysphagia | Aphagia and dysphagia | R13 |
| Incontinence | Fecal incontinence | R15 |
| Infections | Cholera | A00 |
|  | Typhoid and paratyphoid fevers | A01 |
|  | Other salmonella infections | A02 |
|  | Shigellosis | A03 |
|  | Other bacterial intestinal infections (includes *C. difficile*) | A04 |
|  | Other bacterial foodborne intoxications, not elsewhere classified | A05 |
|  | Amebiasis | A06 |
|  | Other protozoal intestinal diseases | A07 |
|  | Viral and other specified intestinal infections | A08 |
|  | Infectious gastroenteritis and colitis, unspecified | A09 |
| Esophageal varices | Esophageal varices | I85 |
| Foreign body in esophagus | Foreign body in esophagus | T18.1 |
| Other | Other diseases of stomach and duodenum | K31 |
|  | Other and unspecified noninfective gastroenteritis and colitis | K52 |
|  | Vascular disorders of intestine | K55 |
|  | Other diseases of intestine | K63 |
|  | Hematemesis, melena, GI hemorrhage | K92 |
|  | Other symptoms and signs involving the digestive system and abdomen | R19 |
|  | Flatulence and related conditions | R14 |

GI, gastrointestinal; GERD, gastroesophageal reflux disease; *Cdiff*, *Clostridioides difficile*.

**Supplementary Table 2.** Comparison of demographics between admitted and discharged patients with gastrointestinal complaints

| Characteristic | Total  (*n* = 62,152,996) | Patients admitted to hospital  (*n* = 10,320,420) | Patients discharged  (*n* = 51,832,576) | *P*-value |
| --- | --- | --- | --- | --- |
| Age, mean ± standard error | 37.9 ± 0.7 | 53.1 ± 1.8 | 34.8 ± 0.7 | < 0.001 |
| Female, *n* (%) | 37,627,517 (60.5) | 5,538,433 (53.7) | 32,089,085 (61.9) | < 0.001 |
| Day of week, *n* (%) |  |  |  | 0.654 |
| Saturday or Sunday | 15,694,928 (25.3) | 2,668,685 (25.9) | 13,026,243 (25.1) |  |
| Weekday | 46,458,068 (74.7) | 7,651,735 (74.1) | 38,806,333 (74.9) |  |
| Residence, *n* (%) |  |  |  | < 0.001 |
| Blank/unknown | 1,054,063 (1.7) | 245,769 (2.4) | 808,294 (1.6) |  |
| Homeless/homeless shelter | 169,757 (0.3) | 63,905 (0.6) | 105,852 (0.2) |  |
| Nursing home | 626,748 (1.0) | 299,123 (2.9) | 327,625 (0.6) |  |
| Other | 419,520 (0.7) | 78,645 (0.8) | 340,875 (0.7) |  |
| Private residence | 59,882,908 (96.3) | 9,632,978 (93.3) | 50,249,930 (96.9) |  |
| Race and ethnicity, *n* (%) |  |  |  | < 0.001 |
| Hispanic | 11,705,986 (18.8) | 1,408,730 (13.6) | 10,297,256 (19.9) |  |
| Non-Hispanic Black | 12,581,705 (20.3) | 1,347,937 (13.1) | 11,233,768 (21.7) |  |
| Non-Hispanic other | 2,056,404 (3.3) | 426,420 (4.1) | 1,629,984 (3.1) |  |
| Non-Hispanic White | 35,808,901 (57.6) | 7,137,333 (69.2) | 28,671,568 (55.3) |  |
| Insurance, *n* (%) |  |  |  | < 0.001 |
| All sources of payment are blank/unknown | 6,134,683 (9.9) | 1,135,829 (11.0) | 4,998,854 (9.7) |  |
| Medicaid or CHIP or other state-based program | 21,522,374 (34.6) | 2,394,360 (23.2) | 19,128,014 (36.9) |  |
| Medicare | 11,369,593 (18.3) | 3,695,196 (35.8) | 7,674,397 (14.8) |  |
| Private insurance | 17,220,178 (27.7) | 2,492,450 (24.2) | 14,727,728 (28.4) |  |
| Worker comp/self-pay/no charge/charity/other | 5,906,168 (9.5) | 602,585 (5.8) | 5,303,583 (10.2) |  |
| CT, *n* (%) |  |  |  | < 0.001 |
| No | 40,823,673 (65.7) | 4,627,169 (44.8) | 36,196,504 (69.8) |  |
| Yes | 21,329,323 (34.3) | 5,693,251 (55.2) | 15,636,072 (30.2) |  |
| MRI, *n* (%) |  |  |  | < 0.001 |
| No | 61,690,398 (99.3) | 10,044,934 (97.3) | 51,645,464 (99.6) |  |
| Yes | 462,598 (0.7) | 275,486 (2.7) | 187,112 (0.4) |  |
| Ultrasound, *n* (%) |  |  |  | 0.018 |
| No | 54,100,019 (87.0) | 8,669,571 (84.0) | 45,430,448 (87.6) |  |
| Yes | 8,052,977 (13.0) | 1,650,849 (16.0) | 6,402,128 (12.4) |  |
| Other imaging, *n* (%) |  |  |  | 0.023 |
| No | 61,469,481 (98.9) | 10,128,740 (98.1) | 51,340,741 (99.1) |  |
| Yes | 683,515 (1.1) | 191,680 (1.9) | 491,835 (0.9) |  |

CHIP, Children’s Health Insurance Program; CT, computed tomography; MRI, magnetic resonance imaging.

**Supplementary Table 3**. Primary discharge diagnoses of the patients in emergency department.

| Diagnosis | Total  (*n* = 62,152,996) | Patients admitted to hospital  (*n* = 10,320,420) | Patients discharged  (*n* = 51,832,576) |
| --- | --- | --- | --- |
| Infections | 1,830,146 (2.94) | 194,212 (1.88) | 1,635,934 (3.16) |
| Abdominal pain | 25,920,455 (41.70) | 2,647,513 (25.65) | 23,272,942 (44.90) |
| Diseases of anus and rectum | 2,032,416 (3.27) | 381,410 (3.70) | 1,651,006 (3.19) |
| Diseases of biliary tract and gallbladder | 1,892,059 (3.04) | 819,055 (7.94) | 1,073,004 (2.07) |
| Diverticular diseases | 1,296,932 (2.09) | 306,506 (2.97) | 990,426 (1.91) |
| Dyspepsia | 26,190 (0.04) | 0 (0.00) | 26,190 (0.05) |
| Dysphagia | 405,617 (0.65) | 163,612 (1.59) | 242,005 (0.47) |
| Esophageal varices | 8,244 (0.01) | 8,244 (0.08) | 0 (0.00) |
| Foreign body in esophagus | 776,566 (1.25) | 234,764 (2.27) | 541,802 (1.05) |
| Functional motility disorders | 3,416,455 (5.50) | 120,419 (1.17) | 3,296,036 (6.36) |
| Gastroesophageal reflux disease and esophageal diseases | 1,478,200 (2.38) | 75,014 (0.73) | 1,403,186 (2.71) |
| Gastrointestinal malignancies | 104,316 (0.17) | 60,761 (0.59) | 43,555 (0.08) |
| Ileus and obstruction | 1,071,976 (1.72) | 880,520 (8.53) | 191,456 (0.37) |
| Incontinence | 1,411 (0.00) | 0 (0.00) | 1,411 (0.00) |
| Liver diseases | 939,681 (1.51) | 550,646 (5.34) | 389,035 (0.75) |
| Malabsorption | 2,222 (0.00) | 2,222 (0.02) | 0 (0.00) |
| Nausea or vomiting | 9,254,224 (14.89) | 858,563 (8.32) | 8,395,661 (16.20) |
| Pancreatic diseases | 1,096,964 (1.76) | 747,467 (7.24) | 349,497 (0.67) |
| Ulcers | 1,770,655 (2.85) | 225,159 (2.18) | 1,545,496 (2.98) |
| Other | 8,828,267 (14.20) | 2,044,333 (19.80) | 6,783,934 (13.09) |
